# Angiogenesis induction in Buerger's disease: a disease management double-edged sword?

**DOI:** 10.1186/s13023-019-1166-6

**Published:** 2019-08-05

**Authors:** Bahare Fazeli, Shayan Keramat, Ladan Assadi, Hossein Taheri

**Affiliations:** 1 Immunology Research Center, Inflammation and Inflammatory Diseases Division, School of Medicine, Mashhad University of Medical Sciences, Mashhad, Iran; 2 Vascular Independent Research and Education, European Foundation, Milan, Italy; 3 Hematology Department, Imam Reza Hospital, Mashhad University of Medical Sciences, Mashhad, Iran; 4 Pathology department, 17Shahrivar Hospital, Mashhad, Iran; 5 Surgery Department, Farabi Hospital, Mashhad, Iran

**Keywords:** Thromboangiitis obliterans, Buerger’s disease, Treatment, Angiogenesis

## Abstract

Due to unknown aetiology of Thromboangiitis obliterans (TAO), its effectively treating is challenging. However, angiogenesis induction is one of the acceptable treatments for TAO patients. Recently, we have noticed that TAO patients who were under long-term treatment with angiogenesis-inducing medication showed considerable improvement in terms of healing chronic ulcers over the course of one to 2 years of treatment. However, some of them developed dermal gangrene despite the warming of their feet, with or without palpable pulses in the extremities, and with hair growth on the affected skin. Unfortunately, following the progression of dermal gangrene, some of these patients had to undergo amputation and limb loss.

During histopathological evaluation, we detected some changes in the amputee TAO patients under long-term angiogenic medical treatment that were not present in amputee TAO patients who had not received any treatment for many years. The greatest pathological changes were observed in the microvascular of the skin, appearing as a proliferation of endothelial cells, NETosis and thrombus formation inside the vessels with proliferation of endothelial cells. The immunohistochemistry for CD31 and Ki67 as markers of vascular endothelium differentiation and cell mitosis confirmed the proliferation of endothelial cells. However, in the patients who had not received any treatment for years the typical pathology view of BD, including preserved vascular architecture with infiltration of inflammatory cells and inflammatory cells inside the thrombus, organised thrombus with recanalisation and intimal thickening was observed. Further longitudinal cohort studies regarding long-term treatment with angiogenic medications for TAO in different geographic areas are highly recommended.

Dear Editor.

Until recently, Thromboangiitis obliterans (TAO) has been regarded as a type of peripheral vascular disease usually involving small and medium-sized arteries [1]. Due to its unknown aetiology, effectively treating TAO is challenging. However, angiogenesis induction is one of the suggestive treatments for TAO for patients with and without critical limb ischemia (CLI). Notably, most of the acceptable medical treatments for TAO that seek to achieve vasodilation can also induce angiogenesis.

For instance, iloprost upregulates the influence of vascular endothelium growth factor (VEGF) on endothelial cell proliferation and angiogenesis [2]. Also, prostaglandin E1 analogues have been reported to induce neovascularisation in ischemic areas mainly by upregulating endothelial nitric oxide synthase (eNOS), hepatocyte growth factor (HGF) and VEGF [3]. Notably, bosentan also induces upregulation of VEGF and eNOS levels in ischemic limbs and induces angiogenesis [4]. Similarly, cilostazol induces angiogenesis and endothelial cell proliferation in ischemic limbs by upregulating HGF and VEGF [5].

Recently, we noted that TAO patients undergoing long-term treatment with angiogenesis-inducing medication, particularly cilostazol, showed considerable improvement in terms of reduced pain and claudication and healing of chronic ulcers over the course of 1 year of treatmesnt.

However, some of them developed dermal gangrene despite the warming of their feet with hair growth on the affected skin and improvement in pain and ulcer healing (Fig. 1). Unfortunately, following the progression of dermal gangrene, some of these patients had to undergo amputation and limb loss.

**Fig. 1:**
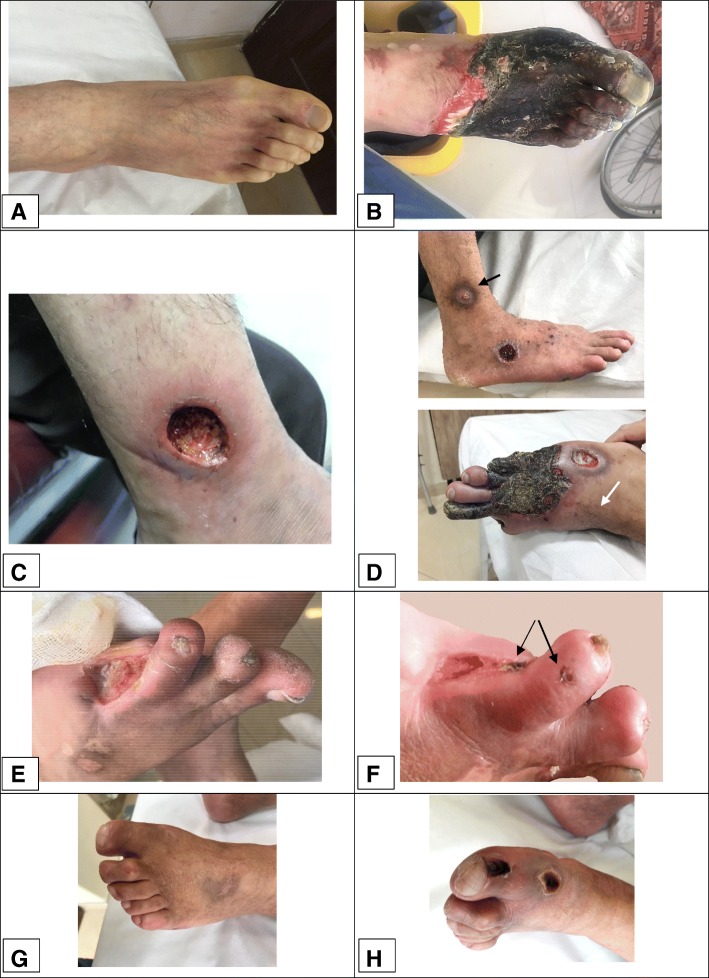
Dermal gangrene in three TAO patients who received long-term angiogenic treatment. **a**, **b** A 30-year-old man with a history of left below-knee amputation due to Buerger’s disease reported to us with severe Raynaud’s phenomenon in the right foot that had lasted for a month. He had undergone treatment with Prostavasin and, because of the dependency of the symptoms for Prostavasin perfusion, he underwent right lumbar sympathectomy, followed by treatment with bosentan. However, the pain diminished, and the foot warmed up for only 3 weeks after sympathectomy. By increasing the pain and discoloration of the foot, the patient received iloprost, followed by cilostazol for 7 months. Although the foot warmed and the dorsalis pedis pulse became palpable, the skin began to experience necrosis, whilst granulation tissue appeared under the dermal gangrene. Due to progression of the gangrene, the patient underwent a second BK amputation. The patient had stopped smoking during the treatment, according to self-report. **c**, **d** A 42-year-old man with an eight-year history of Buerger’s disease reported with a non-healing ulcer on his right ankle and burning pain in the right toes. He received antibiotics as well as cilostazol. The ulcer on the ankle completely healed over the course of 4 months. However, 2 months later, the patient developed a punched-out ulcer on the dorsum of the foot as well as some purpuric-like lesions, which became gangrenous and led to progressive dermal gangrene over the course of 6 months. Finally, the patient underwent below-knee amputation after 1 year of medical treatment. The patient had stopped smoking during the treatment, according to self-report. **e, f:** A 39-year-old man with a four-year history of Buerger’s disease underwent minor amputation and reported with a non-healing ulcer of the amputation stump. He was treated with cilostazol for approximately 2 years, and the ulcer improved. Recently, he developed localised, gangrenous papules and was advised to discontinue cilostazol. He has stopped smoking, according to self-report **g**, **h** A 49-year-old man with a 15-year history of Buerger’s disease began taking cilostazol for claudication. After 1 year, although the claudication had improved, localised dermal gangrene developed, and the cilostazol was discontinued. At present, the gangrene has resolved, but the claudication had progressed. The patient did not stop smoking during the treatment

During histopathological evaluation, we detected some changes in the amputee TAO patients under long-term angiogenic medical treatment that were not present in amputee TAO patients who had not received any treatment for many years.

We studied 68 slides from 29 paraffin blocks of biopsies from two below-knee amputees and two toe amputee patients who were undergoing long-term treatment with antigenic medication. In addition, we evaluated 41 slides from 20 paraffin blocks of biopsies from five below-knee amputees with CLI who underwent amputation without receiving any medical treatment due to the progression of gangrene.

Notably, the gangrene of the extremities was limited to the derma in TAO patients with long-term angiogenic medical treatment, and granulation tissue generation and angiogenesis or a reduction of thrombus formation were observed in the small or medium-sized vessels. However, the greatest pathological changes were observed in the microvascular of the skin, appearing as a proliferation of endothelial cells. NETosis and thrombus formation were also observed inside the vessels with proliferation of endothelial cells (Fig. 2). Immunohistochemistry (IHC) was also performed for cluster of differentiation 31 (CD31) as a marker of vascular endothelium differentiation [6] and antigen Ki67 as a marker of cell mitosis and apoptosis inhibition [7]. According to the results of IHC, extensive proliferation of endothelial cells in the soft tissue and proliferation of endothelial cells in the intima layer of microvessels were observed (Fig. 2).

**Fig. 2:**
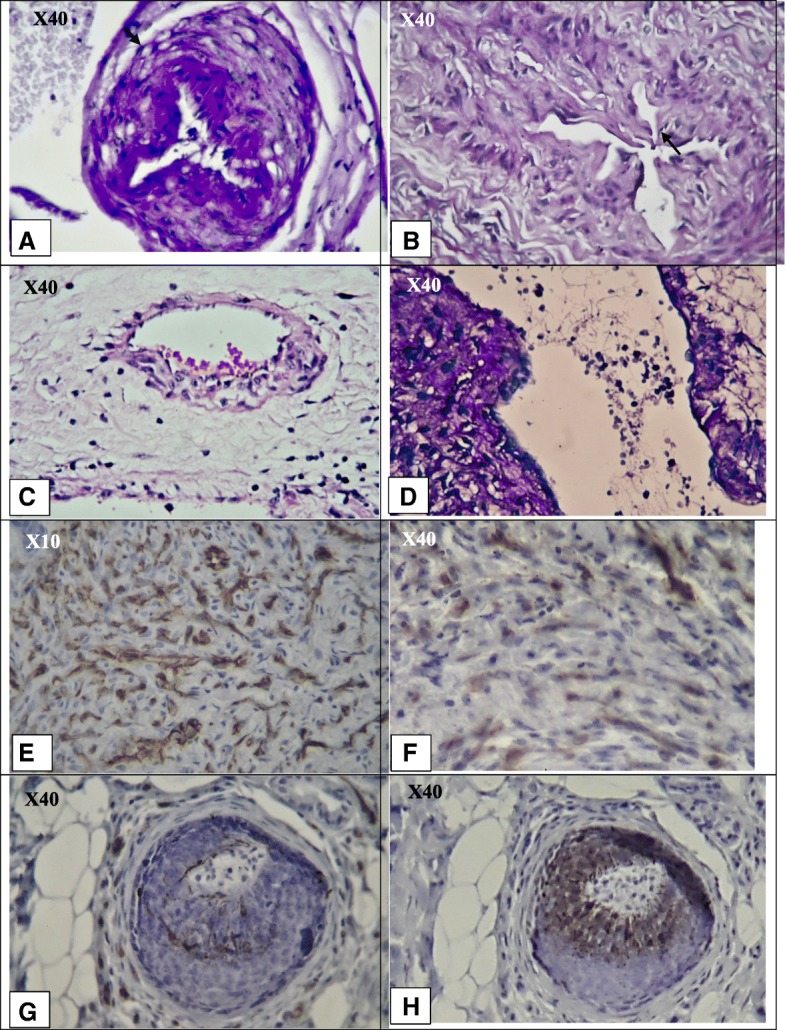
Microvascular changes in the patients with diagnosis of thromboangiitis obliterans with long-term medical angiogenic treatment, according to haemotoxylin and eosin (H & E) and Immunohistochemistry (IHC) for CD31 and Ki67. **a**, **b** h and e staining. X40 objective lense. Stenosis and shrinkage of the microvessel lumen due to proliferation of endothelial cells. **c** **h** and **e** staining. X40 objective lense The proliferation of endothelial cells can be asymmetrical; thrombus formation is at the site of endothelial cell proliferation. **d** **h** and **e** staining. X40 objective lense. Proliferation of endothelial cells can induce NETosis and further thrombus formation. **e** IHC for CD31. X10 objective lense. Extensive proliferation of endothelial cells in the soft tissue. **f** IHC for Ki67. X40 objective lense Mild to moderate positive Ki67 in the soft tissue, which supports the proliferation and mitosis of the endothelial cells.**h** IHC for CD31. X40 objective lense. Proliferation of endothelial cells in the intima layer. Due to the cut, the lumen of the microvessels cannot quite be seen. Instead, part of the stained endothelial cells is seen. **g** IHC for Ki67. X40 objective lense. Supporting the mitosis and proliferation of endothelial cells in the intima layer

However, in the patients who had not received any medical treatment before amputation the typical pathology view of TAO, including preserved vascular architecture with infiltration of inflammatory cells and inflammatory cells inside the thrombus, organised thrombus with recanalisation and intimal thickening was observed. However, proliferation of endothelial cells was not observed, as confirmed by IHC of CD31 and Ki67 (Fig. 3).

**Fig. 3:**
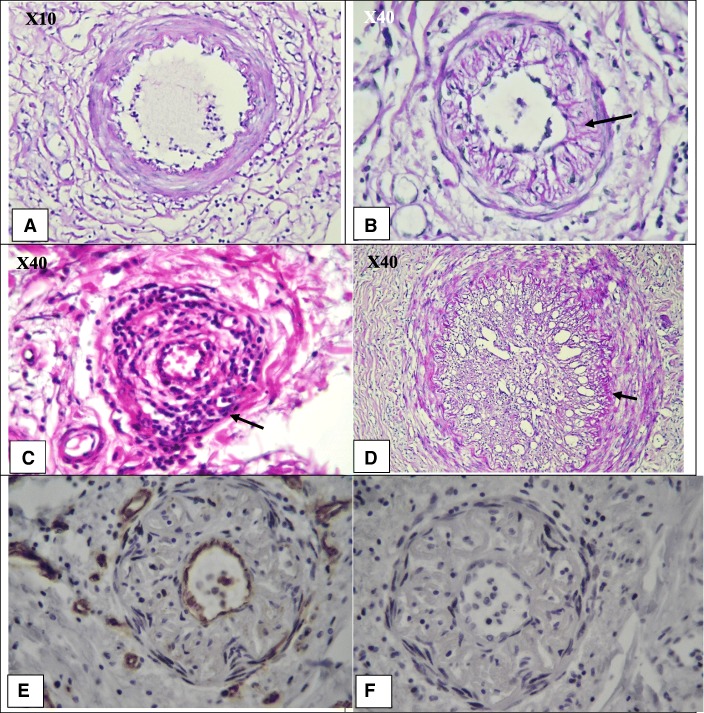
Intimal fibrosis thickening instead of proliferation of endothelial cells, inflammatory thrombus and NETosis formation inside preserved vessels and organised thrombus in a TAO patient who received no treatment for many years. **a** PAS staining. X10 objective lense. Perivascular infiltration of inflammatory cells and the preserved vascular structure of a small artery with early-stage thrombus formation. **b** PAS staining. X40 objective lense Diffuse intimal thiceking with infiltration of inflamamtory cells in the intima layer. **c** H&E staining. X40 objective lense. Infiltration of inflammatory cells in all layers of the vessel. **d** PAS staining. X40 objective lense Organized thrombus with recanalization. The arrow shows basal membrane of intima. **e** IHC for CD31. X40 objective lense. According to the IHC, the intima thickening was not due to the proliferation of endothelial cells. **f** IHC for Ki67. X40 objective lens. Negative results support that intima thickening was not due to any active cell proliferation

It appears that angiogenesis induction in TAO patients through long-term medical treatment, become dysregulated and induced obstruction instead of neo-vascularisation. We noted dermal gangrene primarily in the patients who had been taking cilostazol continually for more than six months. Dermal gangrene can be caused by the angiogenic induction that occurs in long-term use of cilostazol. Notably, Isner et al. also observed that induction of angiogenesis by phVEGF165 gene transfer in TAO patients led to forefoot gangrene in 33% of patients; they ultimately required below-knee amputation, despite evidence of improved perfusion [8].

Whilst the trigger for dysregulated angiogenesis is unknown, long-term treatment with angiogenic medication may be a risk factor for dermal gangrene in TAO patients and ultimately might be a disease management double-edged sword.

All in all, we suggest that TAO patients who receive long-term treatment with angiogenic medication be monitored carefully. Indeed, it might be the better course of action to pause the treatment to observe whether there is any evidence of gangrene. Moreover, a combination of angiogenic and anti-angiogenic medications that can also improve tissue oxygenises, such as pentoxifylline or antioxidants [9], may regulate angiogenesis in TAO and influence the disease outcomes of patients.

The limitation of our study is its small sample size. However, since our findings may influence the limb survival of TAO patients, further longitudinal cohort studies relative to long-term treatment with angiogenic medications in different geographic areas are highly recommended.

## Data Availability

All the pathology slides and blocks in addition to all the pictures from slides of TAO patients are available.

## Abbreviations

eNOS: endothelial nitric oxide synthase
H & E: Haemotoxylin and eosin
HGF: Hepatocyte growth factor
IHC: Immunohistochemistry
NETs: Neutrophil extracellular traps
PAS: Periodic acid–Schiff
TAO: Thromboangiitis obliterans
VEGF: Vascular endothelium growth factors
